# Risk factors and socio-economic burden in pancreatic ductal adenocarcinoma operation: a machine learning based analysis

**DOI:** 10.1186/s12885-020-07626-2

**Published:** 2020-11-27

**Authors:** Yijue Zhang, Sibo Zhu, Zhiqing Yuan, Qiwei Li, Ruifeng Ding, Xunxia Bao, Timing Zhen, Zhiliang Fu, Hailong Fu, Kaichen Xing, Hongbin Yuan, Tao Chen

**Affiliations:** 1 Department of Anesthesiology, South Campus, Renji Hospital, School of Medicine, Shanghai Jiaotong University, Shanghai, China; 2 School of Life Sciences, Fudan University, Shanghai, China; 3 Department of General Surgery, South Campus, Renji Hospital, School of Medicine, Shanghai Jiaotong University, Shanghai, China; 4 School of Medical Instrument and Food Engineering, University of Shanghai for Science and Technology, Shanghai, China; 5 Cinoasia Institute, Shanghai, China; 6 Department of Anesthesiology, Changzheng Hospital, Second Military Medical University, No.415 Fengyang Road, Shanghai, 200003 China; 7 Department of Biliary-Pancreatic Surgery, Renji Hospital, School of Medicine, Shanghai Jiao Tong University, No. 2000 Jiangyue Road, Pujin Street, Minhang District, Shanghai, 201100 China

**Keywords:** Intensive care unit, Machine learning, Risk prediction, Peri-operative, Socio-economic burden, Pancreatic adenocarcinoma

## Abstract

**Background:**

Surgical resection is the major way to cure pancreatic ductal adenocarcinoma (PDAC). However, this operation is complex, and the peri-operative risk is high, making patients more likely to be admitted to the intensive care unit (ICU). Therefore, establishing a risk model that predicts admission to ICU is meaningful in preventing patients from post-operation deterioration and potentially reducing socio-economic burden.

**Methods:**

We retrospectively collected 120 clinical features from 1242 PDAC patients, including demographic data, pre-operative and intra-operative blood tests, in-hospital duration, and ICU status. Machine learning pipelines, including Supporting Vector Machine (SVM), Logistic Regression, and Lasso Regression, were employed to choose an optimal model in predicting ICU admission. Ordinary least-squares regression (OLS) and Lasso Regression were adopted in the correlation analysis of post-operative bleeding, total in-hospital duration, and discharge costs.

**Results:**

SVM model achieved higher performance than the other two models, resulted in an AU-ROC of 0.80. The features, such as age, duration of operation, monocyte count, and intra-operative partial arterial pressure of oxygen (PaO_2_), are risk factors in the ICU admission. The protective factors include RBC count, analgesic pump dexmedetomidine (DEX), and intra-operative maintenance of DEX. Basophil percentage, duration of the operation, and total infusion volume were risk variables for staying in ICU. The bilirubin, CA125, and pre-operative albumin were associated with the post-operative bleeding volume. The operation duration was the most important factor for discharge costs, while pre-lymphocyte percentage and the absolute count are responsible for less cost.

**Conclusions:**

We observed that several new indicators such as DEX, monocyte count, basophil percentage, and intra-operative PaO_2_ showed a good predictive effect on the possibility of admission to ICU and duration of stay in ICU. This work provided an essential reference for indication in advance to PDAC operation.

**Supplementary Information:**

The online version contains supplementary material available at 10.1186/s12885-020-07626-2.

## Background

The current 5-year survival rate of PDAC is only 8% [1], which is the lowest among all common cancers. The incidence of male pancreatic cancer is increasing year by year [1].

Surgical resection is a major way to cure the disease. Adding chemotherapy to adjuvant therapy can improve survival (the five-year survival rate is close to 30%) and reduce peri-operative mortality (about 3%) [2]. However, the risks borne by surgery cannot be underestimated, and the risk of complications is around 50% [3]. The intra-operative risk is mainly bleeding (5.9%), and post-operative complications are mainly pancreatic leakage (13%). These complications may be life-threatening, making the patient’s risk of entering the ICU increased [4, 5]. The high cost of pancreatic cancer surgery directly increases the burden on the patient and family. Patients transferred to the ICU also require special monitoring and intense care. Simultaneously, the number of complications greatly increased the in-hospital cost and length of hospitalization [6]. In a study of emergency department evaluation of suspected ICU patients, it was found that prolonged hospital stay in the emergency department increases the risk of death [7].

Identifying risk factors to predict high-risk groups and correcting surgical procedures has important economic benefits. It is now known that unmodifiable risk factors such as age (> 55), gender (male), blood type (non-O type), modifiable risk factors such as smoking (> 35 cigarettes / d,> 40 years), obesity (BMI > 30) [8, 9]. Accurate peri-operative risk prediction can prevent patients from clinical deterioration, reduce the incidence of adverse events, and control unplanned readmissions to the ICU, mortality, and potentially huge socioeconomic burden. The academic community has found some rules in risk prediction. For example, patients with high-risk surgery have a lower gastric mucosal PHi before surgery. During surgery, tachycardia will make the peri-operative risk higher, increasing the possibility of transfer to ICU [10, 11]. It is still necessary to supplement essential risk factors to assess the risk of postoperative ICU transfer and the incidence of complications to optimize surgical decision-making.

Machine learning (ML) methods have attracted considerable research attention with the development of data storage techniques. ML is a multi-disciplinary interdisciplinary major. It uses computers as a tool and is committed to simulating human learning in real-time, especially how to improve specific algorithms’ performance in empirical learning. ML provides opportunities to improve accuracy by taking advantage of the complex interaction between potential risk factors. It can improve medicine by better exploiting “big data” in a learning way [12]. Studies have shown that machine learning is significantly better than standard clinical reference tools for real-time prediction of complications in intensive care and sepsis prediction [13, 14]. ML can be applied to clinical data sets to develop robust risk models and redefine patient classes [15].

However, few reports on patients’ physiological status before PDAC operation and whether the influencing factors such as pre-operative and intra-operative status or anesthesia intervention will affect the post-operative effect from the real-world and artificial intelligence (AI) angle. Therefore, we collected 120 clinical features from 1242 PDAC patients, including demographic data, pre-operative and intra-operative blood tests, in-hospital duration, and ICU status. After data pre-processing, the 39 filtered variables are finally used for model construction. ML pipelines, including Supporting Vector Machine (SVM), Logistic Regression, and Lasso Regression, were employed to choose an optimal model in predicting ICU admission. Ordinary least-squares regression (OLS) and Lasso Regression were adopted in the correlation analysis of post-operative bleeding, total in-hospital duration, and discharge costs. Establishing a peri-operative risk prediction model helps prevent patients from clinical deterioration and potentially socio-economic burden in advance of the surgery.

## Methods

### Research participants

We retrospectively selected 1242 PDAC patients from existing databases. All participants signed an informed consent form (except for those who have died). The detailed data collection procedure obtained permission from the Ethics Committee of Renji Hospital Affiliated to Shanghai Jiaotong University School of Medicine.

All participants underwent a physical examination and routine blood examination, blood gas analysis, and medication records during the operation, and statistics of clinical and demographic characteristics. Inclusion criteria: patients with pre-operative imaging confirmed pancreatic tumors, with surgical indications, and radical pancreatectomy. Exclusion criteria: The patient did not undergo pancreatic surgery or performed palliative surgery and pancreatic puncture. And those who are confirmed as non-pancreatic primary tumors after the operation (lower bile duct tumors, ampullary tumors, pancreatic metastases, etc.). Criteria for patients being admitted to the ICU are partly referred to ICU admission, discharge, and triage guidelines by Joseph L Nates et al. [16]: peri-operative patients with acute respiratory insufficiency, circulatory instability, severe cardiopulmonary comorbidities, major bleeding, and patients needing life-sustaining interventions.

### Data pre-processing and feature selection

Our structured database initially contains 100 clinical variables. First, features with more than 40% missing were excluded. Then, the categorical variables’ missing values were filled by the mode, and the continuous variables were filled by the random forest [17]. To reduce the influence of the range difference of the features on the model construction, the noncategorical data was processed by mean and SD. Categorical data were further transformed into binary dummy variables. Finally, 39 variables were recruited to build the predictive model for post-operative admission to ICU.

The purpose of feature selection is to determine the best subset of features that can be used to predict each outcome variable. We used the machine learning method lasso regularization to construct feature subsets.

### Model development

**Fig. 1:**
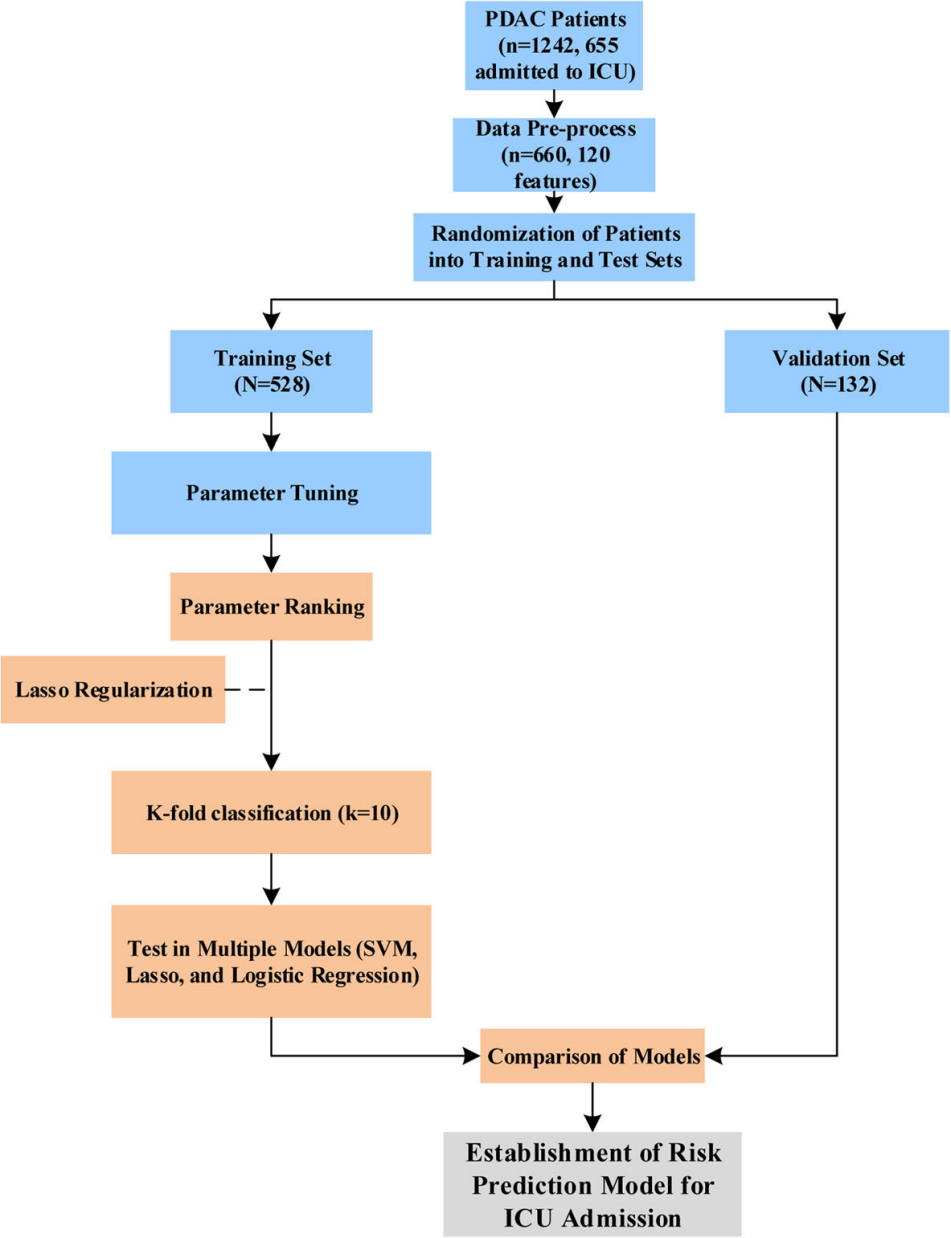
Study flowchart. 1242 patients were recruited in the current study. Through data pre-processing, 660 patients with 120 complete clinical variables were used as predictive variables. The data were pre-processed and randomly divided into a training set (80%) and a validation set (20%). In the training set, k-fold cross-validation (k = 10) is used, and various parameter combinations are exhausted by grid search

Model development includes linear model Lasso [18], Logistic Regression [19] and kernel-based SVM [20] machine learning models. The model was trained in the training set using 10-fold cross-validation, and the grid search method was used to adjust the parameters of each algorithm. In order to quantify the model’s discrimination, a test set was applied to evaluate the model. The categorical dependent variable’s evaluation index includes AU-ROC, sensitivity (recall), specificity, accuracy, log-loss and precision, while the continuous dependent variables are the prediction error graph. In addition, the factor weight of the linear model is taken as the importance of the factor. In addition to the performance comparison, we ranked the effect size of factors contributing to the models. Figure 1 showed the study flowchart.

## Results

### Patients and variables

Our development cohort included a total of 1242 PDAC patients, 665 (52.74%) of whom admission to ICU with a mean time of 16.84 h. Through data pre-processing, 660 patients with 120 complete clinical variables were used as predictive variables (Table 1). ICU admission was considered as an outcome variable to build the predictive model for post-operative admission to ICU. We also built the predictive model for ICU hours, bleeding volume, in-hospital duration, and discharge costs.

**Table 1:** Baseline characteristics of included CRT patients

	Total (*n* = 596)	Responder (*n* = 268)	Non-responder (*n* = 328)	P value
**Demographic characteristics**				
Age	61.67 ± 11.73	62.89 ± 11.04	60.67 ± 12.19	0.02
Male	431 (72.32%)	175 (65.30%)	256 (78.05%)	< 0.01
**Past medical history**				
Ischemic etiology	54 (9.06%)	16 (5.97%)	38 (11.59%)	0.03
Hypertension	241 (40.44%)	118 (44.03%)	123 (37.50%)	0.13
Diabetes mellitus	110 (18.46%)	55 (20.52%)	55 (16.77%)	0.29
Atrial fibrillation	121 (20.30%)	47 (17.54%)	74 (22.56%)	0.16
Prior CIED implantation	46 (7.72%)	16 (5.97%)	30 (9.15%)	0.20
History of SCD	67 (11.24%)	24 (8.96%)	43 (13.11%)	0.14
History of PCI	41 (6.88%)	10 (3.73%)	31 (9.45%)	< 0.01
History of CABG	6 (1.01%)	0 (0.00%)	6 (1.83%)	0.04
**Clinical status**				
NYHA class	2.80 ± 0.67	2.75 ± 0.66	2.84 ± 0.68	0.11
Weight (kg)	66.32 ± 12.22	65.78 ± 12.11	66.78 ± 12.31	0.36
SBP (mmHg)	117.40 ± 18.28	119.93 ± 19.38	115.31 ± 17.07	< 0.01
DBP (mmHg)	73.81 ± 10.37	74.35 ± 10.59	73.36 ± 10.17	0.25
**Biochemical tests**				
Hemoglobin (g/L)	133.46 ± 17.22	132.91 ± 16.96	133.92 ± 17.44	0.48
Lymphocytes (%)	27.19 ± 9.43	28.05 ± 9.50	26.48 ± 9.33	0.04
RDW-CV (%)	13.82 ± 2.51	13.59 ± 3.14	14.00 ± 1.82	0.06
RDW-SD (fL)	46.48 ± 17.40	44.76 ± 4.94	47.88 ± 22.95	0.02
Total bilirubin (μmol/L)	16.42 ± 10.62	14.82 ± 9.58	17.73 ± 11.25	< 0.01
Combined bilirubin (μmol/L)	7.18 ± 6.73	6.17 ± 6.03	8.01 ± 7.16	< 0.01
Albumin (g/L)	40.40 ± 5.02	40.91 ± 5.45	39.97 ± 4.59	0.03
Alanine transaminase (U/L)	28.43 ± 34.30	24.61 ± 18.52	31.58 ± 42.94	< 0.01
Aspartate transaminase (U/L)	26.97 ± 23.23	23.91 ± 11.60	29.49 ± 29.34	< 0.01
Blood urea nitrogen (mmol/L)	7.79 ± 3.61	7.50 ± 3.49	8.02 ± 3.70	0.05
Serum creatinine (μmol/L)	93.48 ± 33.25	90.76 ± 30.98	95.74 ± 34.90	0.07
eGFR (ml/min/1.73 m^2^)	74.82 ± 24.36	75.21 ± 22.38	74.50 ± 25.92	0.72
Serum uric acid (μmol/L)	447.64 ± 133.83	427.14 ± 126.73	464.59 ± 137.33	< 0.01
Fasting glucose (mmol/L)	5.87 ± 2.07	6.01 ± 2.28	5.75 ± 1.87	0.14
Total cholesterol (mmol/L)	4.13 ± 1.00	4.14 ± 1.00	4.13 ± 1.00	0.91
Sodium (mmol/L)	140.30 ± 3.97	140.72 ± 3.95	139.96 ± 3.95	0.02
Creatine kinase (U/L)	84.18 ± 88.84	80.40 ± 59.78	87.34 ± 107.25	0.34
Creatine kinase-MB (U/L)	12.43 ± 4.97	12.33 ± 4.51	12.50 ± 5.33	0.68
C-reactive protein (mg/L)	8.19 ± 16.46	7.19 ± 15.84	8.91 ± 16.90	0.27
Hemoglobin A1c (%)	6.34 ± 1.13	6.36 ± 1.22	6.32 ± 1.05	0.71
cTnT (ng/ml)	0.06 ± 0.14	0.06 ± 0.15	0.06 ± 0.12	0.60
NT-proBNP (pg/ml)	3867.71 ± 4795.63	3049.77 ± 3734.94	4521.55 ± 5415.93	< 0.01
Free triiodothyronine (pmol/L)	4.09 ± 0.83	4.17 ± 0.78	4.02 ± 0.86	0.04
Free thyroxine (pmol/L)	17.97 ± 3.48	17.46 ± 3.17	18.40 ± 3.67	< 0.01
TSH (uIU/ml)	3.62 ± 5.44	3.12 ± 3.22	4.03 ± 6.72	0.04
**Electrocardiographic parameters**				
Atrial fibrillation	109 (18.29%)	43 (16.04%)	66 (20.12%)	0.24
QRS morphology				< 0.01
LBBB	391 (65.60%)	213 (79.48%)	178 (54.27%)	
RBBB	40 (6.71%)	5 (1.87%)	35 (10.67%)	
IVCD	133 (22.32%)	38 (14.18%)	95 (28.96%)	
Paced	30 (5.03%)	11 (4.10%)	19 (5.79%)	
QRS duration (ms)	163.95 ± 23.94	166.17 ± 21.66	162.14 ± 25.54	0.04
RR interval (ms)	847.10 ± 202.88	826.47 ± 189.64	863.88 ± 211.84	0.02
Corrected QT interval (ms)	488.54 ± 46.90	495.35 ± 47.49	483.00 ± 45.75	< 0.01
**Echocardiographic parameters**				
LAD (mm)	49.54 ± 8.42	47.09 ± 7.84	51.53 ± 8.37	< 0.01
LVEDD (mm)	69.37 ± 10.01	67.40 ± 8.61	70.99 ± 10.76	< 0.01
LVESD (mm)	58.36 ± 10.17	55.96 ± 9.25	60.32 ± 10.48	< 0.01
IVS (mm)	9.33 ± 2.01	9.33 ± 1.85	9.32 ± 2.14	0.95
LVPW (mm)	9.30 ± 1.68	9.33 ± 1.62	9.28 ± 1.73	0.72
PAP (mmHg)	42.51 ± 15.34	39.95 ± 13.30	44.58 ± 16.54	< 0.01
LVEF (%)	31.51 ± 7.25	31.22 ± 6.68	31.75 ± 7.68	0.37
MR	2.50 ± 0.93	2.38 ± 0.96	2.61 ± 0.89	< 0.01
TR	1.69 ± 0.96	1.56 ± 0.87	1.80 ± 1.03	< 0.01
**Medication at discharge**				
Diuretics	525 (88.09%)	230 (85.82%)	295 (89.94%)	0.16
ACEI	354 (59.40%)	158 (58.96%)	196 (59.76%)	0.91
ARB	176 (29.53%)	86 (32.09%)	90 (27.44%)	0.18
ARNI	2 (0.34%)	1 (0.37%)	1 (0.30%)	1.00
β-blocker	530 (88.93%)	240 (89.55%)	290 (88.41%)	0.76
Spironolactone	536 (89.93%)	245 (91.42%)	291 (88.72%)	0.34
Ivabradine	99 (16.61%)	50 (18.66%)	49 (14.94%)	0.27
Digoxin	153 (25.67%)	53 (19.78%)	100 (30.49%)	< 0.01
Amiodarone	103 (17.28%)	27 (10.07%)	76 (23.17%)	< 0.01
Statin	155 (26.01%)	70 (26.12%)	85 (25.91%)	1.00
Warfarin	77 (12.92%)	30 (11.19%)	47 (14.33%)	0.31

*ACEI* angiotensin converting enzyme inhibitor, *ARB* angiotensin II receptor blocker, *ARNI* angiotensin receptor-neprilysin inhibitor, *CABG* coronary artery bypass grafting, *CIED* cardiac implantable electronic device, *cTnT* cardiac troponin T, *DBP* diastolic blood pressure, *eGFR* estimated glomerular filtration rate, *IVCD* non-specific interventricular conduction delay, *IVS* interventricular septum thickness, *LAD* left atrial diameter, *LBBB* left bundle branch block, *LVEDD* left ventricular end-diastolic diameter, *LVEF* left ventricular ejection fraction, *LVESD* left ventricular end-systolic diameter, *LVPW* left ventricular posterior wall thickness, *MR* mitral regurgitation, *NT-proBNP* N-terminal prohormone of brain natriuretic peptide, *PCI* percutaneous coronary intervention, *RBBB* right bundle branch block, *RDW-CV* red blood cell distribution width (RDW) -coefficient of variation, *RDW-SD* RDW-standard deviation, *SBP* systolic blood pressure, *TR* tricuspid regurgitation, *TSH* thyroid stimulating hormone

### Validation of training set for post-operative evaluation of ICU

**Fig. 2:**
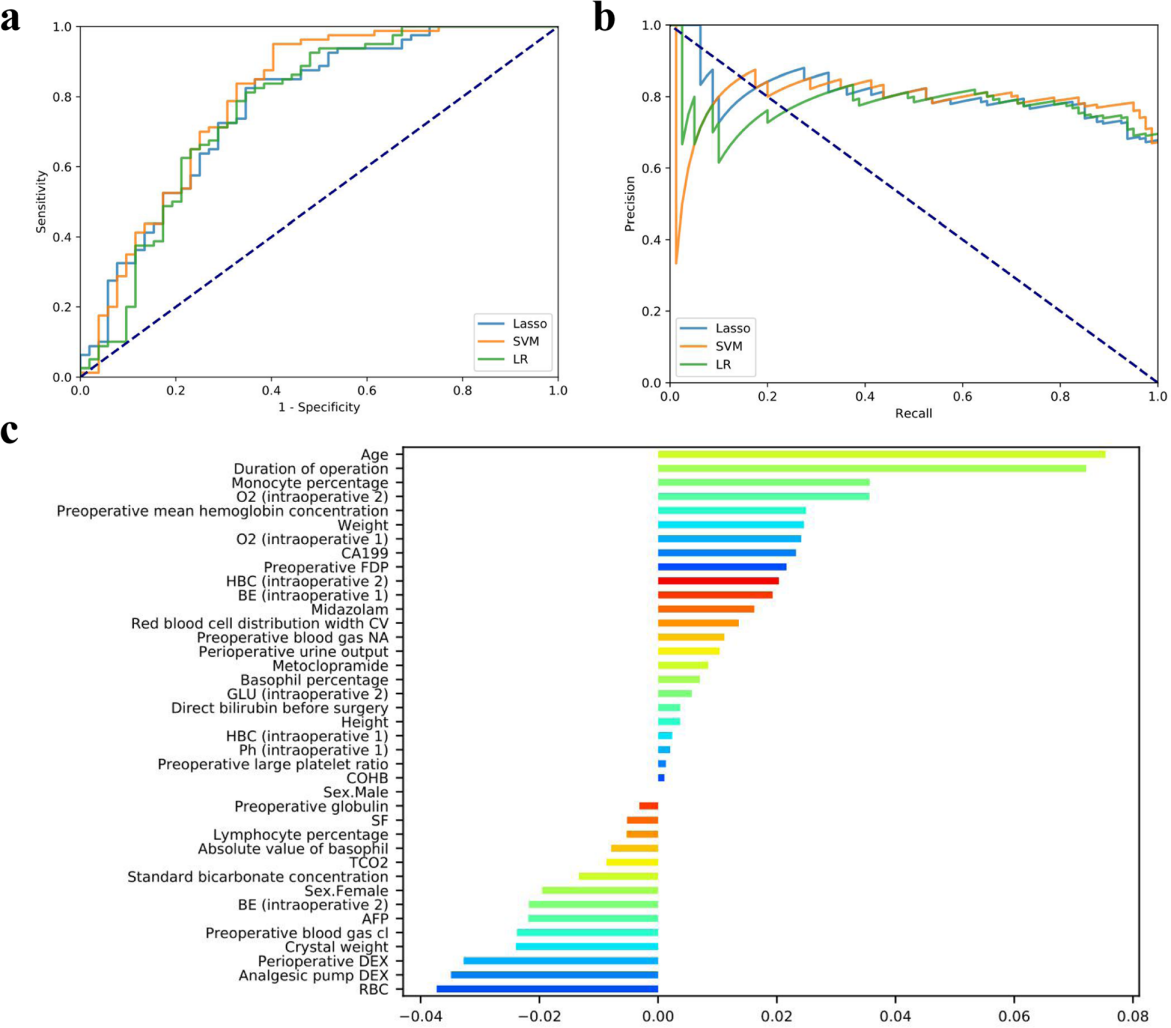
Evaluation of the predictive models. **a** The average ROC curves from of three models in the validation sets. **b** The average PR curves, indicating the tradeoff between precision and recall. **c** The histogram describes the importance features of the predictive model for post-operative admission to ICU

The average ROC curves and PR curves was shown in the predictive model establishment of ICU admission in three models, i.e., SVM, Lasso, and LR (Fig. 2a and 1b). All models have AUC values above 0.75, and the SVM is present to be upper (0.80). We use the A*P* value as the criterion for the PR curve. It can be seen that the APs of SVM and Lasso models are all above 0.80. The confusion matrix (rounding) was also calculated for these models (Table 2). SVM generates the minimum number of FN (4) during the prediction process. The model LR produced the minimum number of FP (18).

**Table 2:** Confusion matrices of post-operative evaluation-ICU

Model	Actual	Predictive	
		Negative	Positive
SVM	Negative	31	4
	Positive	21	76
Lasso	Negative	33	12
	Positive	19	68
LR	Negative	34	15
	Positive	18	65

Table 3 showed the AUC, Sensitivity, Specificity, Accuracy, log-loss, FP Rate, Precision, AP, and F1 of each model evaluation result. There are significant performance differences between the different models. All models have excellent performance, and the accuracy rate is up to 0.75. Among them, SVM obtains the highest AUC value of 0.80, and the accuracy rate is 0.81. The Lasso has an AU-ROC value of 0.77, and the accuracy rate is 0.77. LR obtains the lowest AUC value of 0.76, and the accuracy rate is 0.75. The best performance of Sensitivity is the model SVM, which is suitable for the predictive model for post-operative admission to ICU in patients with PDAC. The model SVM, Lasso, and LR’s Sensitivity reached over 0.80, and the specificity rate is over 0.60. SVM performed best in FP Rate and Precision.

**Table 3:** Performance summary of post-operative evaluation-ICU

Models	AUC	95%CI		sensitivity (recall)	specificity	accuracy	log-loss	FP rate	precision	AP	F1
		Lower bound	Upper bound								
SVM	0.80	0.71	0.88	0.95	0.60	0.81	0.53	0.4	0.78	0.8	0.86
Lasso	0.77	0.68	0.86	0.85	0.63	0.77	0.56	0.37	0.78	0.81	0.81
LR	0.76	0.67	0.85	0.81	0.65	0.75	0.56	0.35	0.69	0.78	0.75

Feature importance was calculated by the sum of the decrease in error when split by a variable, reflecting each variable’s contribution to ICU admissions. The important features of the predictive model for post-operative admission to ICU, as were shown in effect sizes, were calculated, as shown in Fig. 2c. The features, such as age, duration of operation, monocyte, O_2_ (intra-operative), and pre-operative mean hemoglobin concentration et al., are risk factors. The protective factors include RBC, analgesic pump DEX, intra-operative DEX, crystal weight, and pre-operative blood gas Cl et al. (Fig. 2c and Supplementary Figure 1).

### Predictive model for post-operative evaluation of ICU hours and intra-operative bleeding volume

**Fig. 3:**
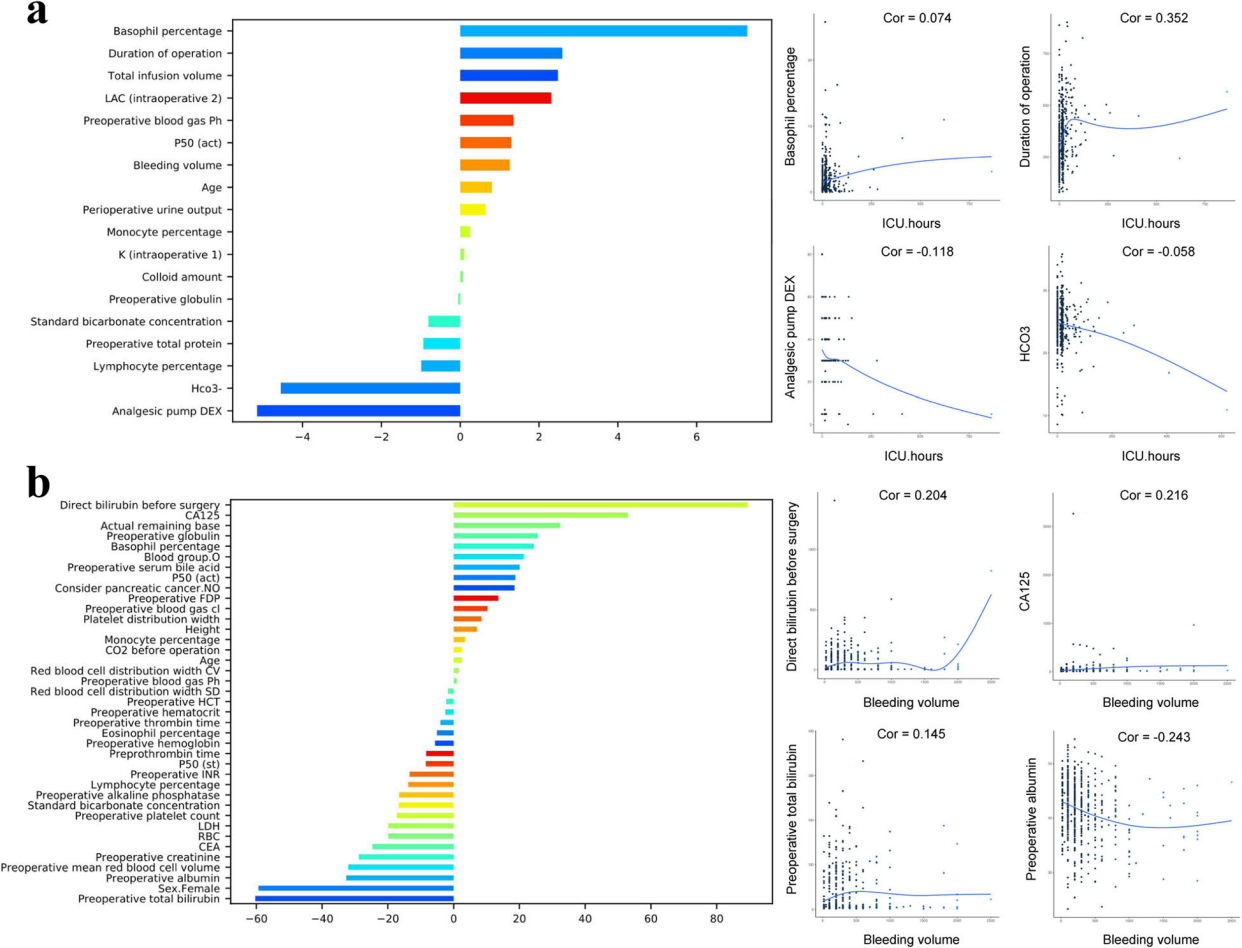
**a** The importance features of the predictive model for post-operative evaluation of ICU hours. **b** The importance features of the predictive model for intra-operative bleeding volume

The basophil percentage was the most important risk variable for post-operative evaluation of ICU hours, followed by the duration of the operation and total infusion volume. The protective factors include analgesic pump DEX, HCO3, and lymphocyte percentage et al. The higher feature value of direct bilirubin before surgery, CA125, and actual remaining base increased probability of intra-operative bleeding volume, and pre-operative total bilirubin, Sex. Female and pre-operative albumin decreased bleeding volume probability (Fig. 3).

### Predictive model for evaluation of in-hospital duration and discharge costs

**Fig. 4:**
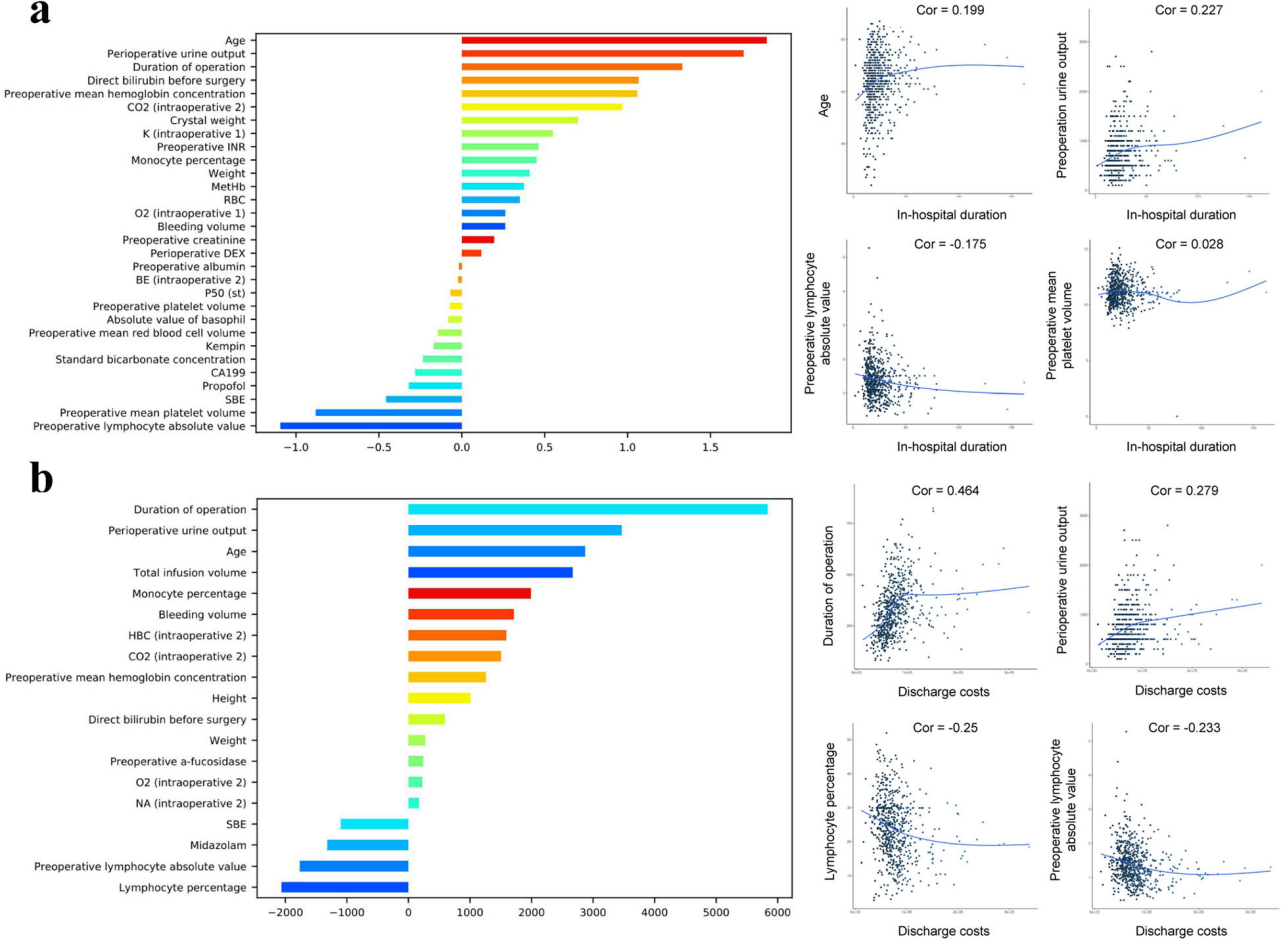
**a** The importance features of the predictive model evaluation of in-hospital duration. **b** The importance features of the predictive model for discharge costs

The risk factors for post-operative evaluation of in-hospital duration were age, pre-operation urine output, and operation duration. The protective factors were Pre-operative lymphocyte absolute value, Pre-operative mean platelet volume, and SBE. The operation duration was the most important risk variable for discharge costs, followed by peri-operative urine output, age, and total infusion volume. The protective factors for discharge costs include lymphocyte percentage, pre-operative lymphocyte absolute value, and midazolam (Fig. 4).

## Discussion

In our study, we compared and developed machine learning models to predict ICU admission, bleeding volume, in-hospital duration, and discharge costs by collecting 1242 patients with PDAC surgery and recording 120 pre-operative, intra-operativ and post-operative variables. Logistic regression is a generalized linear model that converts nonlinear factors through the sigmoid function to handle classification problems well. Yihe Wu et al. used logistic regression to analyze risk factors significantly associated with postoperative pulmonary complications (PPCs) in patients undergoing minimally invasive lobectomy [21]. The results showed that both restrictive and liberal intraoperative fluid administration were related to adverse effects on postoperative outcomes. Lasso is a linear regression method using L1 regularization, which will make some of the learned feature weights zero, to achieve sparseness and feature selection. Tadahiro Goto et al. applied regularization methods such as lasso and ridge to predict the disposition of asthma and COPD exacerbations in the ED to avoid overfitting when machine learning predicts complex relationships [22]. The learning algorithm of SVM is the optimal algorithm for solving convex quadratic programming. Abeg Kumar Jaiswal et al. were based on the SVM classification algorithm for automatic EEG seizure detection, which had obtained good prediction results and revealed the application potential of SVM in other prediction fields [23]. In our ICU admission prediction test, the SVM model achieved higher performance than other models, resulted in an AU-ROC of 0.80.

Feras Hawari et al.’s study identified smoking status and having received chemotherapy were potentially modifiable risk factors for ICU admission of patients with cancer [24]. 50% of ICU admissions were due to sepsis and respiratory failure, some of which were cancer-specific [25, 26]. Another study reported that the role of key physiologic variables in predicting ICU admissions. For example, Hampshire et al. showed that tachypnea and hypotension were associated with increased hospital mortality [27]. Williams et al.’s study showed that factors such as age, duration of operation, and various complications could determine admission to an ICU [28]. The result is the same as our study. Besides, we also found other factors such as PaO_2_ level, monocyte count, and DEX that affect ICU admissions.

The PaO_2_ value of the radial artery was monitored as routine in our study. As patients intake pure oxygen (inspired oxygen of 100%) in operation, the normal PaO_2_/FiO_2_ values are usually above 300 mmHg, according to ALI and ARDS diagnosis guidelines [29]. However, in our study, we found that intra-operative PaO_2_ was a risk factor to enhancing the post-operative ICU admission incidence rate. There have been reports of direct and indirect adverse effects of oxygen in the perioperative period [30].

Studies have shown that a higher death rate was observed in the high-flow oxygen group than the titrated oxygen group in COPD patients [31]. The same situation reported that intake of 100% oxygen after 15 min of cerebral ischemia for 3 to 6 h significantly increased the 14-day mortality to three-fold compared to the air intake group [32].

Meanwhil, intaking high-flow oxygen to the post-operative patient, especially high-risk patients, will not prevent reintubation or extubation of respiratory failure [33]. Studies also indicated that hyperoxia could cause acute lung injury and impairment of lung function [34, 35], which was considered an independent factor associated with in-hospital mortality [36]. Mechanisms of hypoxia-induced injury mainly induced oxidative stress, which may activate necroptosis. Hypoxia can cause an imbalance of non-inflammatory factors and anti-inflammatory factors in the lungs through various ways, resulting in the release of inflammatory factors in the lungs and causing damage; it can affect the initiation of NF-kb in macrophages and aggravate the initiation of the inflammatory response; in addition, hypoxia can cause increased production of ROS induced lung damage. Combined with the ‘duration of operation’, the PaO_2_ increase the rick to ICU admission is easy to understand. In our study, it is worth noted that the PaO_2_ in the PDAC operation should be controlled at a relatively lower level to guarantee a lower ICU admission incidence rate. These findings suggest that the high flow of oxygen or high PaO_2_ status during surgery is not beneficial to patients, but rather impaired lung function, especially in patients with pre-operative pulmonary insufficiency, such as COPD, increasing the likelihood of failure post-operative extubation and increased the risk to ICU. Therefore a more well-designed clinical trial should be performed to validate the hypothesis.

Monocyte is a risk factor in predicting ICU admissions. There have also been some previous studies on the relationship between monocyte and PDAC, suggesting that high monocyte in pancreatic cancer patients is usually suggestive of shorter survival and poor prognosis [37–39], which can serve as in independent factor to predict the survival of pancreatic cancer with resection [37]. In particular, monocytes appear to play an important role in determining patient outcomes following surgery [40]. Although the prognosis and survival of PDAC patients were not addressed in this part of our study, monocytes play an important role in tumor proliferation and metastasis. They are also associated with tumor-induced systemic inflammatory responses. We hypothesized that an increase in monocytes predicted that the whole body was already undergoing an inflammatory response. After undergoing greater surgical stimulation, the inflammatory response’s exacerbation caused damage to vital organs such as the heart, lungs, and brain. However, the characterization of the early post-operative immune response in ICU patients with a causal link to later post-operative infections lacks from the current literature.

Dexmedetomidine is a highly selective α2-adrenergic receptor agonist, providing sedative and analgesic effects without respiratory depression. Studies found that DEX could inhibit the inflammatory response. In human studies, Dex could reduce the release of serum inflammatory markers CRP, TNF-α, IL-6, and IL-1β, which indicated a strong effect on anti-inflammatory reaction [41]. Further studies confirm that dextromethorphan may provide lung protection through various pathways, such as attenuating pulmonary ischemia-reperfusion injury through the PI3K/Akt/HIF-1α signaling pathway [42], protecting lung tissue by modulating immune responses [43], and also providing pulmonary protection from hyperoxia induced lung injury by attenuated the ROS [44]. In clinical, DEX is a popular medicine used for sedation in the ICU. DEX can provide safe and effective sedation, facilitate extubation, and reduce delirium, atrial fibrillation, and renal and myocardial injury [45]. DEX was necessary to prevent post-operative complications from pre-admission interventions for older cardiac surgery patients [46]. In our study, the feature of analgesic pump DEX and intra-operative DEX are protective factors for ICU admissions, probably due to the anti-inflammatory effect, which against the high monocyte and protective effect against the high PaO_2_ induced injury.

ICU length of stay (LOS) is a frequent measure of ICU resource use and performance. Predictions of ICU LOS are routinely used as the means of resource allocation. However, the accuracy of ICU LOS predictions made by clinicians has been poorly evaluated. Studies reported that variables, such as post-operative monitoring, systolic arterial pressure, creatinine level, invasive mechanical ventilation, and active infection et al., were associated with ICU LOS [47–50]. In the ICU time-length study, Huang performed two types of analyses, in which a single-factor correlation analysis found a large number of changes in the blood cell indexes of hospitalized patients in relation to their in-hospital mortality, both mentioning the ratio of monocytes to basophils; in the multifactorial regression, basophils, leukocytes, MCHC were independent factors associated with in-hospital mortality [51]. After analyzing the data through artificial intelligence and deep learning, Our results only suggested that basophil percentage was a potential risk variable for post-operative evaluation of ICU LOS, as an independent fast associated with the in-hospital mortality.

In addition, we analyzed the characteristics of the potential factors of intra-operative bleeding volume and in-hospital duration. We found that the in-hospital duration was related to pre-operative urine volume, lymphocyte absolute value. The bilirubin, CA125, and pre-operative albumin were associated with bleeding volume, which were rarely reported in previous studies. The location of the pancreatic tumor often determines its clinical presentation, such as direct bilirubin. Direct bilirubin elevation in our study can increase in intraoperative bleeding, most likely related to tumor oppression, resulting in increased bleeding due to increased surgical difficulty. However, more research is needed to explain the increase in surgical bleeding caused by Ca125 and the findings that preoperative total bilirubin can reduce surgical bleeding.

In our study, age, operative time, PaO_2_, and monocyte were found to be risk factors for increased ICU entry in the model predicting ICU stay; in the model predicting ICU stay, basophils percentage, duration of operation, total infusion volume were found to be risk factors for increased ICU stay; in the model predicting ICU stay, age, operative urine volume, and direct preoperative bilirubin were found to be risk factors for increased ICU stay; in the model predicting operative expense summary, we found operative time, urine volume, age, and total infusion to be major risk factors.

Within this range of predictive models and factors, except for age as the recognized risk factor, other factors and models directly still have some potential linkage. For example, the “direct bilirubin before surgery” response is the degree of obstruction of the biliary tract system by the pancreatic tumor, which directly affects the difficulty of surgery and causes the increase in the operating time, increasing the probability of entering the ICU, the length of stay in the ICU and the medical expenses. And the prolonged duration of the operation also caused an increase in intraoperative urine volume and total infusion volume, which has to be reflected in other predictive models. Besides, the prolonged duration of surgery increases the time to high PaO2, causing damage to vital organs and increasing the risk of patients entering the ICU.

There are no standard criteria for admission to the ICU in different regions and hospitals. In our study, the criteria for admission to the ICU were based on the clinical experience of the current hospital, in addition to the criteria of the surgeon and anesthesiologist. The high-risk factors identified in our predictive models, in addition to alerting and assisting surgeons and anesthesiologists in clinical decisions, can also serve to provide data support for future ICU admission criteria for pancreatic cancer patients in the future. The ultimate goal is to provide effective advice and standards for access to the ICU and rationalize medical resources allocation through the continuous expansion of data volume and the enrichment of clinical disease types, which was the purpose of Nates’ study, published in 2016 in the journal *Critical Care Medicine* [16].

For patients with PDAC, survival, morbidity, and sequelae are significant and necessary outcome indicators. Since many patients in our database were operated on from 2018 to 2019, the best observation period of long-term results (such as a 3-year survival period) has not yet been reached, so it has not been analyzed in this study. Patients’ outcome is also of great concern and interest to us, and we will further analyze it in the follow-up study.

## Conclusions

In conclusion, we developed a machine learning model to predict ICU admission in this study. There are essential values for reducing patients’ financial burden and provides new clinical insights for improving peri-operative management of PDAC patients.

## Supplementary Information

**Additional file 1 **: **Supplementary figure 1**. The top features of the predictive model for post-operative admission to ICU.

## Data Availability

The datasets used and/or analysed during the current study are available from the corresponding author on reasonable request.

## Abbreviations

PDAC: pancreatic ductal adenocarcinoma
ICU: intensive care unit
SVM: Supporting Vector Machine
OLS: Ordinary least-squares regression
PaO2: partial arterial pressure of oxygen
DEX: dexmedetomidine
ML: Machine learning
AI: artificial intellegence
PPCs: postoperative pulmonary complications
LOS: length of stay

## References

[1] Siegel RL, Miller KD, Jemal A (2018). Cancer statistics, 2018. CA Cancer J Clin.

[2] Strobel O, Neoptolemos J, Jager D, Buchler MW (2019). Optimizing the outcomes of pancreatic cancer surgery. Nat Rev Clin Oncol.

[3] Smits FJ, Henry AC, van Eijck CH, Besselink MG, Busch OR, Arntz M, Bollen TL, van Delden OM, van den Heuvel D, van der Leij C (2020). Care after pancreatic resection according to an algorithm for early detection and minimally invasive management of pancreatic fistula versus current practice (PORSCH-trial): design and rationale of a nationwide stepped-wedge cluster-randomized trial. Trials.

[4] Harnoss JC, Ulrich AB, Harnoss JM, Diener MK, Buchler MW, Welsch T (2014). Use and results of consensus definitions in pancreatic surgery: a systematic review. Surgery.

[5] Smits FJ, van Santvoort HC, Besselink MG, Batenburg MCT, Slooff RAE, Boerma D, Busch OR, Coene P, van Dam RM, van Dijk DPJ (2017). Management of Severe Pancreatic Fistula after Pancreatoduodenectomy. JAMA Surg.

[6] Bateni SB, Olson JL, Hoch JS, Canter RJ, Bold RJ (2018). Drivers of cost for pancreatic surgery: It's not about hospital volume. Ann Surg Oncol.

[7] Zhang Z, Bokhari F, Guo Y, Goyal H (2019). Prolonged length of stay in the emergency department and increased risk of hospital mortality in patients with sepsis requiring ICU admission. Emerg Med J.

[8] Midha S, Chawla S, Garg PK (2016). Modifiable and non-modifiable risk factors for pancreatic cancer: a review. Cancer Lett.

[9] Bosetti C, Lucenteforte E, Silverman DT, Petersen G, Bracci PM, Ji BT, Negri E, Li D, Risch HA, Olson SH (2012). Cigarette smoking and pancreatic cancer: an analysis from the international pancreatic Cancer case-control consortium (Panc4). Ann Oncol.

[10] Poeze M, Takala J, Greve JW, Ramsay G (2000). Pre-operative tonometry is predictive for mortality and morbidity in high-risk surgical patients. Intensive Care Med.

[11] Hartmann B, Junger A, Rohrig R, Klasen J, Jost A, Benson M, Braun H, Fuchs C, Hempelmann G (2003). Intra-operative tachycardia and peri-operative outcome. Langenbeck's Arch Surg.

[12] Obermeyer Z, Emanuel EJ (2016). Predicting the future - big data, machine learning, and clinical medicine. N Engl J Med.

[13] Meyer A, Zverinski D, Pfahringer B, Kempfert J, Kuehne T, Sundermann SH, Stamm C, Hofmann T, Falk V, Eickhoff C (2018). Machine learning for real-time prediction of complications in critical care: a retrospective study. Lancet Respir Med.

[14] Desautels T, Calvert J, Hoffman J, Jay M, Kerem Y, Shieh L, Shimabukuro D, Chettipally U, Feldman MD, Barton C (2016). Prediction of Sepsis in the intensive care unit with minimal electronic health record data: a machine learning approach. JMIR Med Inform.

[15] Deo RC (2015). Machine learning in medicine. Circulation.

[16] Nates JL, Nunnally M, Kleinpell R, Blosser S, Goldner J, Birriel B, Fowler CS, Byrum D, Miles WS, Bailey H (2016). ICU admission, discharge, and triage guidelines: a framework to enhance clinical operations, development of institutional policies, and further research. Crit Care Med.

[17] Stekhoven DJ, Bühlmann P (2011). MissForest—non-parametric missing value imputation for mixed-type data. Bioinformatics.

[18] Tibshirani R (1996). Regression shrinkage and selection via the lasso. J R Stat Soc B..

[19] Stoltzfus JC (2011). Logistic regression: a brief primer. Acad Emerg Med.

[20] Burges CJ (1998). A tutorial on support vector machines for pattern recognition. Data Min Knowl Disc.

[21] Wu Y, Yang R, Xu J, Rusidanmu A, Zhang X (2019). Effects of Intraoperative Fluid Management on Postoperative Outcomes After Lobectomy. Ann Thoracic Surg..

[22] Tadahiro G, Camargo CA, Kamal FM, Yun BJ, Kohei H (2018). Machine learning approaches for predicting disposition of asthma and COPD exacerbations in the ED. Am J Emerg Med..

[23] Jaiswal AK, Banka H (2018). Epileptic seizure detection in EEG signal using machine learning techniques. Australas Phys Eng Sci Med.

[24] Hawari FI, Nazer LH, Addassi A, Rimawi D, Jamal K (2016). Predictors of ICU admission in patients with Cancer and the related characteristics and outcomes: a 5-year registry-based study. Crit Care Med.

[25] Soares M, Caruso P, Silva E, Teles JM, Lobo SM, Friedman G, Dal Pizzol F, Mello PV, Bozza FA, Silva UV (2010). Characteristics and outcomes of patients with cancer requiring admission to intensive care units: a prospective multicenter study. Crit Care Med.

[26] Azoulay E, Mokart D, Pene F, Lambert J, Kouatchet A, Mayaux J, Vincent F, Nyunga M, Bruneel F, Laisne LM (2013). Outcomes of critically ill patients with hematologic malignancies: prospective multicenter data from France and Belgium--a groupe de recherche respiratoire en reanimation onco-hematologique study. J Clin Oncol.

[27] Hampshire PA, Welch CA, McCrossan LA, Francis K, Harrison DA (2009). Admission factors associated with hospital mortality in patients with haematological malignancy admitted to UK adult, general critical care units: a secondary analysis of the ICNARC case mix Programme database. Crit Care.

[28] Williams TA, Dobb GJ, Finn JC, Knuiman MW, Geelhoed E, Lee KY, Webb SA (2008). Determinants of long-term survival after intensive care. Crit Care Med.

[29] Papazian L, Aubron C, Brochard L, Chiche JD, Combes A, Dreyfuss D, Forel JM, Guerin C, Jaber S, Mekontso-Dessap A (2019). Formal guidelines: management of acute respiratory distress syndrome. Ann Intensive Care.

[30] Lumb AB, Walton LJ (2012). Perioperative oxygen toxicity. Anesthesiol Clin.

[31] Austin MA, Wills KE, Blizzard L, Walters EH, Wood-Baker R (2010). Effect of high flow oxygen on mortality in chronic obstructive pulmonary disease patients in prehospital setting: randomised controlled trial. BMJ.

[32] Mickel HS, Vaishnav YN, Kempski O, von Lubitz D, Weiss JF, Feuerstein G (1987). Breathing 100% oxygen after global brain ischemia in Mongolian gerbils results in increased lipid peroxidation and increased mortality. Stroke.

[33] Hernandez G, Vaquero C, Colinas L, Cuena R, Gonzalez P, Canabal A, Sanchez S, Rodriguez ML, Villasclaras A, Fernandez R (2016). Effect of Postextubation high-flow nasal cannula vs noninvasive ventilation on Reintubation and Postextubation respiratory failure in high-risk patients: a randomized clinical trial. JAMA.

[34] Bhandari V, Choo-Wing R, Lee CG, Zhu Z, Nedrelow JH, Chupp GL, Zhang X, Matthay MA, Ware LB, Homer RJ (2006). Hyperoxia causes angiopoietin 2-mediated acute lung injury and necrotic cell death. Nat Med.

[35] Carnesecchi S, Deffert C, Pagano A, Garrido-Urbani S, Metrailler-Ruchonnet I, Schappi M, Donati Y, Matthay MA, Krause KH, Barazzone Argiroffo C (2009). NADPH oxidase-1 plays a crucial role in hyperoxia-induced acute lung injury in mice. Am J Respir Crit Care Med.

[36] Kilgannon JH, Jones AE, Shapiro NI, Angelos MG, Milcarek B, Hunter K, Parrillo JE, Trzeciak S, Emergency medicine shock research network I (2010). Association between arterial hyperoxia following resuscitation from cardiac arrest and in-hospital mortality. JAMA.

[37] Sanford DE, Belt BA, Panni RZ, Mayer A, Deshpande AD, Carpenter D, Mitchem JB, Plambeck-Suess SM, Worley LA, Goetz BD (2013). Inflammatory monocyte mobilization decreases patient survival in pancreatic cancer: a role for targeting the CCL2/CCR2 axis. Clin Cancer Res.

[38] Baj-Krzyworzeka M, Szatanek R, Weglarczyk K, Baran J, Urbanowicz B, Branski P, Ratajczak MZ, Zembala M (2006). Tumour-derived microvesicles carry several surface determinants and mRNA of tumour cells and transfer some of these determinants to monocytes. Cancer Immunol Immunother.

[39] Zhou W, Ke SQ, Huang Z, Flavahan W, Fang X, Paul J, Wu L, Sloan AE, McLendon RE, Li X (2015). Periostin secreted by glioblastoma stem cells recruits M2 tumour-associated macrophages and promotes malignant growth. Nat Cell Biol.

[40] Torrance HDT, Longbottom ER, Vivian ME, Lalabekyan B, Abbott TEF, Ackland GL, Hinds CJ, Pearse RM, O'Dwyer MJ (2018). Post-operative immune suppression is mediated via reversible, Interleukin-10 dependent pathways in circulating monocytes following major abdominal surgery. PLoS One.

[41] Flanders CA, Rocke AS, Edwardson SA, Baillie JK, Walsh TS (2019). The effect of dexmedetomidine and clonidine on the inflammatory response in critical illness: a systematic review of animal and human studies. Crit Care.

[42] Zhang W, Zhang JQ, Meng FM, Xue FS (2016). Dexmedetomidine protects against lung ischemia-reperfusion injury by the PI3K/Akt/HIF-1alpha signaling pathway. J Anesth.

[43] Song Q, Lin L, Chen L, Cheng L, Zhong W (2020). Co-administration of N-acetylcysteine and dexmedetomidine plays a synergistic effect on protection of LPS-induced acute lung injury via correcting Th1/Th2/Th17 cytokines imbalance. Clin Exp Pharmacol Physiol.

[44] Sifringer M, von Haefen C, Krain M, Paeschke N, Bendix I, Buhrer C, Spies CD, Endesfelder S (2015). Neuroprotective effect of dexmedetomidine on hyperoxia-induced toxicity in the neonatal rat brain. Oxidative Med Cell Longev.

[45] Karaman Y, Abud B, Tekgul ZT, Cakmak M, Yildiz M, Gonullu M (2015). Effects of dexmedetomidine and propofol on sedation in patients after coronary artery bypass graft surgery in a fast-track recovery room setting. J Anesth.

[46] Ettema RG, Van Koeven H, Peelen LM, Kalkman CJ, Schuurmans MJ (2014). Preadmission interventions to prevent postoperative complications in older cardiac surgery patients: a systematic review. Int J Nurs Stud.

[47] Nassar AP, Caruso P (2016). ICU physicians are unable to accurately predict length of stay at admission: a prospective study. Int J Qual Health Care.

[48] Toptas M, Sengul Samanci N, Akkoc I, Yucetas E, Cebeci E, Sen O, Can MM, Ozturk S (2018). Factors affecting the length of stay in the intensive care unit: our clinical experience. Biomed Res Int.

[49] Bohmer AB, Just KS, Lefering R, Paffrath T, Bouillon B, Joppich R, Wappler F, Gerbershagen MU (2014). Factors influencing lengths of stay in the intensive care unit for surviving trauma patients: a retrospective analysis of 30,157 cases. Crit Care.

[50] Ohta Y, Sakuma M, Koike K, Bates DW, Morimoto T (2014). Influence of adverse drug events on morbidity and mortality in intensive care units: the JADE study. Int J Qual Health Care.

[51] Huang YL, Hu ZD (2016). Lower mean corpuscular hemoglobin concentration is associated with poorer outcomes in intensive care unit admitted patients with acute myocardial infarction. Ann Transl Med.

