# Supplementary material for: Risk factors and socio-economic burden in pancreatic ductal adenocarcinoma operation: a machine learning based analysis
Source: BMC Cancer. 2020 Nov 27;20:1161. doi: 10.1186/s12885-020-07626-2 (PMC7694304; doi:10.1186/s12885-020-07626-2)
Supplement: Supplementary file 1 — Additional file 1 : Supplementary figure 1. The top features of the predictive model for post-operative admission to ICU. [file 12885_2020_7626_MOESM1_ESM.docx]

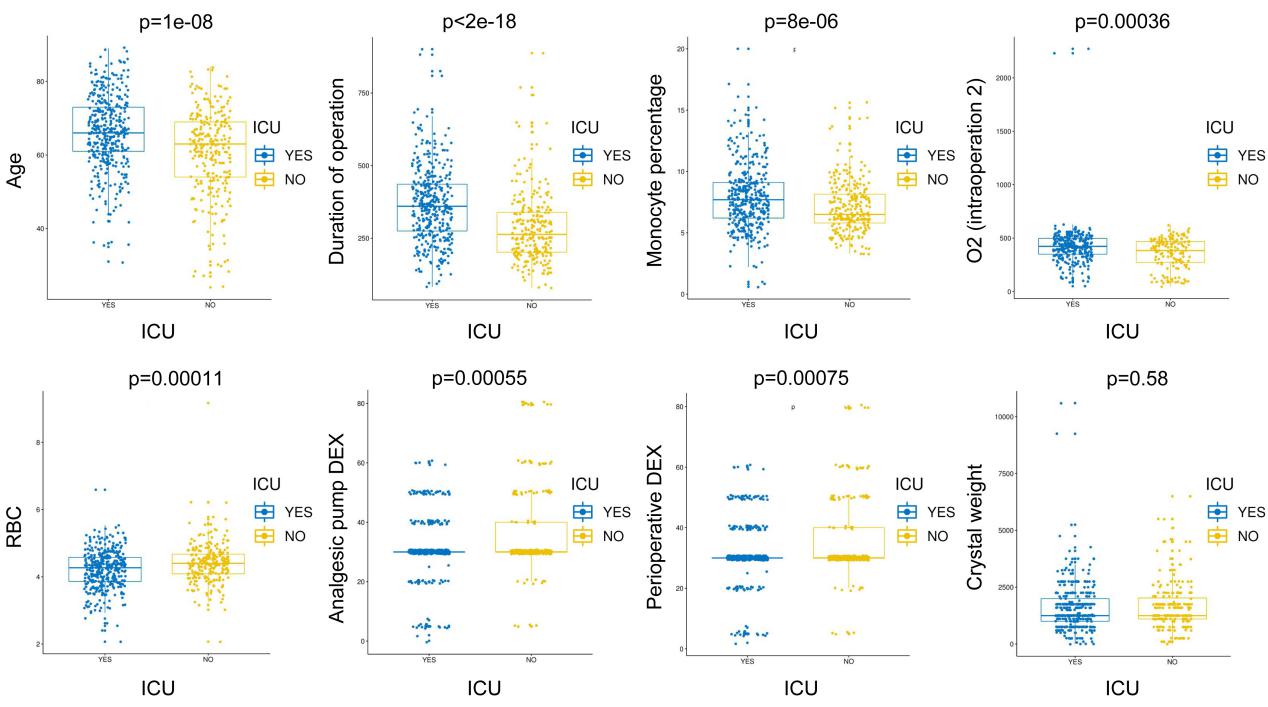


**Supplementary figure 1.** The top features of the predictive model for post-operative admission to ICU.
